# Diagnostic Role of mNGS in Polymicrobial Periprosthetic Joint Infection

**DOI:** 10.3390/jcm12051838

**Published:** 2023-02-24

**Authors:** Jian Mei, Hongxin Hu, Si Zhu, Haiqi Ding, Zida Huang, Wenbo Li, Bin Yang, Wenming Zhang, Xinyu Fang

**Affiliations:** 1 Department of Orthopaedic Surgery, The First Affiliated Hospital, Fujian Medical University, Fuzhou 350005, China; 2 Department of Orthopaedics, The Affiliated Hospital of Putian University, Putian 351100, China; 3 Department of Orthopaedic Surgery, National Regional Medical Center, Binhai Campus of the First Affiliated Hospital, Fujian Medical University, Fuzhou 350212, China; 4 Fujian Provincial Institute of Orthopedics, The First Affiliated Hospital, Fujian Medical University, Fuzhou 350005, China; 5 Department of Laboratory Medicine, The First Affiliated Hospital, Fujian Medical University, Fuzhou 350005, China

**Keywords:** diagnosis, metagenomic next-generation sequencing, periprosthetic joint infection, polymicrobial

## Abstract

Objectives: The purpose of this study was to explore the clinical value of metagenomic next-generation sequencing (mNGS) in the diagnosis of polymicrobial periprosthetic joint infection (PJI). Methods: Patients with complete data who underwent surgery at our hospital between July 2017 and January 2021 for suspected periprosthetic joint infection (PJI), according to the 2018 ICE diagnostic criteria, were enrolled, and all patients underwent microbial culture and mNGS detection, which were performed on the BGISEQ-500 platform. Microbial cultures were performed on two samples of synovial fluid, six samples of tissue, and two samples of prosthetic sonicate fluid for each patient. The mNGS was performed on 10 tissues, 64 synovial fluid samples, and 17 prosthetic sonicate fluid samples. The results of mNGS testing were based on the interpretation of mNGS results in the previous literature and the assertions of microbiologists and orthopedic surgeons. The diagnostic efficacy of mNGS in polymicrobial PJI was assessed by comparing the results of conventional microbial cultures and mNGS. Results: A total of 91 patients were finally enrolled in this study. The sensitivity, specificity, and accuracy of conventional culture for the diagnosis of PJI were 71.0%, 95.4%, and 76.9%, respectively. The sensitivity, specificity, and accuracy of mNGS for the diagnosis of PJI were 91.3%, 86.3%, and 90.1%, respectively. The sensitivity, specificity, and accuracy of conventional culture for the diagnosis of polymicrobial PJI were 57.1%, 100%, and 91.3%, respectively. mNGS had a sensitivity, specificity, and accuracy of 85.7%, 60.0%, and 65.2%, respectively, for the diagnosis of polymicrobial PJI. Conclusions: mNGS can improve the diagnosis efficiency of polymicrobial PJI, and the combination of culture and mNGS is a promising method to diagnose polymicrobial PJI.

## 1. Introduction

Periprosthetic Joint Infection (PJI) is one of the serious complications after joint replacement. The literature reports that the incidence of PJI is 0.5–3% for initial replacement and 4–6% for revision [1]. The majority of PJIs are caused by a single pathogen, but some are still multiple infections caused by two or more pathogens [2]. The incidence of polymicrobial PJI has been noted to be 6–37% [3,4,5,6,7]. Some of the pathogens are often present as polymicrobial infections, such as *Enterococcus faecalis* [8], Gram-negative bacilli [9], etc.

The outcome of PJI treatment often depends on the characteristics of the pathogen, including the virulence of the pathogen and its drug resistance [4]. Many clinical studies have shown that polymicrobial PJI tends to have worse clinical outcomes compared to monomicrobial PJI [3,10,11]. Inadequate diagnosis of polymicrobial infection may result in ineffective postoperative antibiotic therapy, and may lead to the failure of revised surgery. At present, traditional microbial culture methods for the diagnosis of polymicrobial infections have limitations. Many bacteria have competitive inhibition effects in the culture [12], which leads to missed diagnoses; indolent bacteria or low-virulent microorganisms are difficult to culture and identify [10]. Some specific pathogens, such as *Mycoplasma*, often require special culture methods [13], and are often missed clinically. The microbial culture is the gold standard for the diagnosis of bone and joint infections [14], and even with extended culture times, optimized culture conditions, and so on, some cultures fail to detect pathogenic bacteria [15]. It has been noted that the culture remains negative in 7–12% of cases, even though other indicators of infection are present [16], at a rate as high as 41% in the case report [17].

In recent years, molecular diagnostic techniques have been applied to the diagnosis of PJI. These techniques include culture-independent techniques, which can detect nucleic acids in clinical samples within hours [18]. 16S rRNA/rDNA PCR is currently the accepted method for the identification of pathogens, however, it is still unable to identify fungal or polymicrobial infections [19]. Multiplex PCR (mPCR) makes it possible to detect several pathogens in one test and is capable of detecting pathogens within 2 h, but with a limited number of targets (<20), and microarray methods and PCR mass spectrometry are able to detect more microorganisms [20,21], but the target probes or primers are predefined and difficult to update [22]. In addition, the detection time for microorganisms is critical and important for treatment decision-making, while traditional culture methods take longer to detect pathogenic bacteria, reaching 45 days for *Mycobacterium* [23].

The Next-Generation Sequencing (NGS) technology, also known as high-throughput or massive parallel sequencing, can simultaneously sequence thousands of DNA fragments. NGS, including metagenomics Next-Generation Sequencing (mNGS), has the advantage of the unbiased detection of pathogens, and can reduce the time from sample receipt to final results from 48 h to 6 h with nanopore-sequencing technology [23,24]. In particular, the advantages of mNGS are more pronounced in detecting *Mycobacterium tuberculosis* (MTB), viruses, anaerobes, and fungi. Furthermore, mNGS was less affected by prior antibiotic exposure [25]. The application of mNGS in PJI has shown a diagnostic sensitivity of over 90% [26,27]. However, there are few studies on the use of mNGS in the diagnosis of polymicrobial infections. mNGS was tested in mixed pulmonary infections by Wang et al. [28], but its application in polymicrobial PJI is unclear.

Therefore, this study mainly had the following aims: (1) To observe the diagnostic performance of mNGS in diagnosing polymicrobial PJI diagnosis. (2) To observe the combined application of mNGS and conventional microbial culture in clinical practice.

## 2. Methods

### 2.1. Study Population Selection

This study was approved by the Ethics Committee of the First Affiliated Hospital of Fujian Medical University ([2015]084-2). In this retrospective study, patients with suspected PJI who underwent surgery at our center between July 2017 and January 2021 were sequentially enrolled. The diagnosis of PJI was based on 2018 ICM diagnostic criteria for PJI [29]. Inclusion criteria were as follows: (1) Patients who were suspected of having PJI, based on medical history, physical examination or imaging, and who eventually underwent surgery; (2) Those who had sufficient specimens (tissue, synovial fluid, or prosthetic sonicate fluid) for culture and mNGS testing. Exclusion criteria were as follows: (1) clinical and laboratory data were incomplete for a diagnosis of PJI or aseptic failure (AF); (2) specimens were contaminated or suspected of contamination; (3) sequencing failed due to sample quality problems. The demographic characteristic, medical history, physical signs, serum inflammatory indexes, synovial fluid white blood cells count (SF-WBC), the percentage of polymorphonuclear cells of the synovial fluid (SF-PMN%), conventional microbial culture results, and mNGS results were recorded.

### 2.2. Specimen Collection and Processing

We collected intraoperative samples, including tissue, synovial fluid, and ultrasound fluid, from PJI suspected patients. During the operation, synovial fluid was obtained by puncture, after skin incision and before the incision of the joint capsule, avoiding contamination caused by exposure of the incision; 6 tissue samples were taken from each patient, and the surgical assistants immediately cut them into pieces under aseptic conditions and packed them into a closed sterile container for transfer; prostheses were removed and immediately packed in a sterilized closed container for sonication. The specific methods were described in our previous reports [27,30,31]. Specimens were transferred to the microbiology laboratory for microbial culture within half an hour after collection. A total of 0.1 mL aliquots of joint fluid and ultrasound-treated fluid were incubated on blood agar (Thermo Fisher Scientific, Waltham, MA, USA) at 35–37 °C, 5–7% CO_2_ for 7 and 14 days under aerobic and anaerobic conditions, respectively. Residual samples were inoculated into BACTEC Peds Plus/F bottles and incubated in a BD automated incubator (Becton-Dickinson, Heidelberg, Germany) under aerobic and anaerobic conditions for 5 and 14 days, respectively, and if the results were positive, they were passaged on blood agar. Tissues were homogenized in broth (Sangon Biotech, Shanghai, China) and inoculated according to the protocol described above, with every sample plated in triplicate. All bacterial identifications were performed using the Vitek 2 line (BioMerieux Vitek, Inc., Cambridge, MA, USA). The rest of the specimens were stored at −80 °C for mNGS testing to avoid contamination.

### 2.3. mNGS

The mNGS was performed according to the previously described method [32]. The main steps are as follows: (1) nucleic acid extraction: 500 µL of liquid or homogenized tissue was taken, and total DNA was extracted using the TIANamp Micro DNA Kit (DP316, Tiangen, Beijing, China), according to the instructions of the reagents. (2) Library construction and sequencing was performed. DNA was randomly split into 200–300 bp fragments and the concentration of DNA libraries was detected using the dsDNA HS Assay Kit (Thermo Fisher Scientific, USA). After cyclization, the libraries were replicated by rolling cycles to generate DNA nanospheres. The prepared DNA nanospheres were loaded onto sequencing chips and sequenced using the BGISEQ-500 platform (UWIC, Changshu, China). (3) Bioinformatics analysis: low-quality data and data smaller than 35 bp were removed, and then sequenced by BWA (Burrows-Wheeler alignment, v 0.7.17, http://bio-bwa.sourceforge.net, accessed on 1 January 2021). The human reference genome sequence (Hg19) was removed by comparison. The remaining data were compared with microbial databases and then classified as viruses, fungi, bacteria, parasites, etc. The microbial reference whole-genome data included 1494 bacteria, 2700 viruses, 73 fungi, 40 mycoplasma or chlamydia bacterial species, and 48 parasites, all from the National Center for Biotechnology Information (https://ncbi.nlm.nih.gov/genome, accessed on 1 January 2021) [30,33].

### 2.4. Interpretation of mNGS Results

The interpretation of mNGS results is based on previous literature and our previous study [25,30,34,35,36]. The genome coverage rate (CR) was defined as the length of the detected pathogen sequences divided by the total length of the reference genome. Relative abundance at the genus level was defined as the proportion of microbial genera in the same broad class (bacteria, fungi, viruses, parasites) among the detected pathogens. Combined with previous reports in the literature, the thresholds for detection were set as follows: (1) *Burkholderia*, *Ralstonia*, *Delftia*, *Sphingobium*, *Alternaria*, *Sodaria*, *Aspergillus*, *Albugo*, and other genera were the most common background bacteria, measured within other sample species in our laboratory. The pathogenic bacteria were identified when their relative abundance at the genus level was ≥80%. (2) The pathogenic genera with the highest CR and standardized number of reads stringently mapped to pathogen in the species level (SDSMRNS) were identified as pathogenic bacteria. (3) The optimal threshold for bacterial identification was determined to be ≥15% relative abundance at the level of non-human genera and ≥30% relative abundance at the level of fungal genera. (4) Due to the extremely low number of nucleic acids, the *Mycobacterium tuberculosis* complex was identified as a pathogen after normalization to the number of reads that were stringently mapped to the pathogens at the genus level, with (SDSMRNG) ≥1.

### 2.5. Clinical Diagnostic Criteria for Polymicrobial PJI

**Figure 1:**
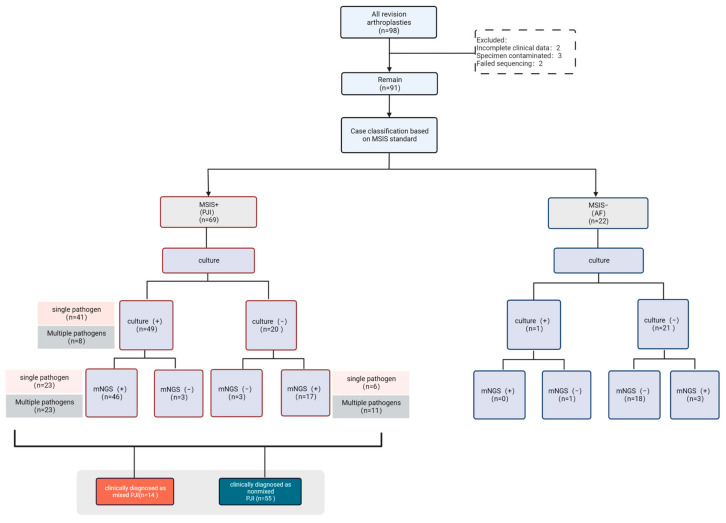
Diagnostic flow chart for polymicrobial PJI.

The patients included in this study were diagnosed with PJI or AF by 2018 ICM diagnostic criteria [29], and then PJI cases were judged to have a polymicrobial PJI based on the following criteria: (1) according to mNGS and culture results (the two results may be inconsistent), it is confirmed that the detected multiple pathogens (two or more) were the causative agents reported in the previous literature; (2) The antibiotic regimen was made by at least two orthopedic specialists (WZ, WL, XF) and a microbiologist (BY) based on the pathogens detected, and the infection was cured. The diagnostic process of polymicrobial PJI is shown in Figure 1.

### 2.6. Statistical Analysis

Differences between PJI and AF were assessed using the Chi-squared test or Fisher’s exact test for categorical variables, and the Mann–Whitney U-test for non-normally distributed parameters. Sensitivity, specificity, Positive Predictive Rate (PPV), Negative Predictive Rate (NPV) and accuracy were calculated for each diagnostic method. McNemar’s Chi-squared test (two-sided) was used to compare the sensitivity and specificity of the diagnostic tests. All analyses were performed using EmpowerStats software v 3.0 (www.empowerstats.net, accessed on 1 January 2021).

## 3. Results

### 3.1. Demographic Characteristics

The demographic characteristics and clinical manifestations of the patients are shown in Table 1. According to the inclusion criteria, ninety-eight patients were included, two cases were excluded due to insufficient clinical data, three cases were obviously contaminated with specimens, and two cases failed to sequence. Finally, a total of 91 patients were included, including 38 males and 53 females, and 46 hips and 45 knees, with a median age of 64.6 ± 13.5 (IQR 58 to 72). According to the MSIS criteria, sixty-nine patients (75.8%) were finally diagnosed with PJI, including 27 males and 42 females, with a median age of 65.3 ± 13.5 (IQR 59.5 to 73.2), and including 30 hips and 39 knees. Twenty-two patients (24.2%) were diagnosed with AF, including eleven males and eleven females; the median age was 62.7 ± 13.6 (IQR 57.2 to 70.5), with 14 hips and 8 knees. There were no differences in age, sex, or joint involvement between the two groups. Fifteen patients in the PJI group had sinus tracts and twenty-three patients had preoperative antibiotic use, whereas none in the AF group. The CRP, ESR, SF-WBC count, and SF-PMN% were higher in the PJI group than in the AF group and were significantly different (Mann-Whitney U-test, *p* < 0.001). 

### 3.2. Comparison of mNGS and Conventional Microbial Culture in Pathogens Detection

#### 3.2.1. Culture Results

We routinely performed bacterial cultures on two samples of synovial fluid, six samples of tissue, and two samples of ultrasound lysate for each case, and a total of fifty cases ended up with positive culture results. According to conventional microbial culture results, there were a total of 22 cases in the AF group; one case was positive, and no pathogen was detected in the other twenty-one cases. In the PJI group, 49 cases were culture-positive and 20 cases (29%) were culture-negative. Culture-negative and culture-positive patients were defined as CN-PJI, and CP-PJI, respectively. 

#### 3.2.2. mNGS Results

Of the 91 cases undergoing mNGS, there were 10 tissues, 64 synovial fluids, and 17 ultrasonic lysates, and a total of 66 cases ultimately had positive mNGS results. In the AF group, mNGS detected pathogens in three cases, and nineteen were negative. Among 20 CN-PJIs, mNGS analysis produced negative results in 3 cases (15%), and produced positive detection in 17 cases, of which 6 had monomicrobial results and 11 had polymicrobial results. Of the the 49 CP-PJIs, mNGS analysis produced negative results in 3 cases, produced monomicrobial detection in 23 cases and polymicrobial detection in another 23 cases. In conclusion, in general, mNGS is superior to conventional microbial culture in pathogen detection.

### 3.3. Comparison of Diagnostic Efficacy of Conventional Microbial Culture and mNGS

The comparison of mNGS and conventional microbial culture for PJI diagnosis is presented in Table 2. The sensitivity, NPV, and diagnostic accuracy of conventional microbial culture were lower than the mNGS method (Table 2), while the specificity and PPV of conventional microbial culture were higher than the mNGS method (Table 2).

### 3.4. Diagnostic Efficacy of Culture and mNGS for Polymicrobial PJI Diagnosis

The comparison of mNGS and conventional microbial culture for polymicrobial PJI diagnosis is presented in Table 3. At present, there is no clear “gold standard” for the diagnosis of polymicrobial PJI. In this study, a clinical diagnosis of polymicrobial PJI is used as the standard, and a polymicrobial infection was defined when two or more pathogens were identified. The specificity, PPV, and diagnostic accuracy of conventional microbial culture for the diagnosis of polymicrobial PJI were higher than those of mNGS (Table 3), while the sensitivity and NPV of conventional microbial culture for the diagnosis of polymicrobial PJI were lower than those of mNGS (Table 3). 

### 3.5. Case Analysis of Clinically Diagnosed Polymicrobial Infection

Although mNGS produced multiple pathogens detection in 34 cases, only 14 cases were identified as polymicrobial PJI by the aforementioned clinical diagnostic criteria. All cases were treated with corresponding antibiotics and cured according to the pathogens identified by culture and mNGS. The detailed data are shown in Table 4. Among these cases, three were acute PJI and eleven were chronic PJI, and two were CN-PJI and twelve were CP-PJI, of which four cultures had monomicrobial results, and eight had polymicrobial results. The details are as follows:

### 3.6. Cases with Negative Culture Result but Diagnosed as Polymicrobial Infection after Supplemental Pathogen Information by mNGS

Of the 14 cases clinically diagnosed as polymicrobial infection, two cases (Case 3 and 12) were CN-PJI with a negative culture result, and mNGS complemented the detection of certain pathogens. In Case 3, mNGS detected *Mycoplasma hominis* and *Acinetobacter baumannii*. In Case 12, mNGS detected *Pseudomonas aeruginosa*, *Klebsiella oxytoca*, and *Mycoplasma hominis*. In conclusion, mNGS improved the efficiency of polymicrobial PJI diagnosis in culture-negative cases.

### 3.7. Cases with Single Pathogens Cultured but Diagnosed as Polymicrobial Infection after Supplemental Pathogen Information by mNGS 

Among the 14 cases clinically diagnosed as polymicrobial infection, 4 cases (Case 2, 5, 7 and 14) were identified, by conventional microbial culture, to have only a single pathogen. In these four cases, mNGS produced multiple pathogens detection in two cases (Case 7 and 14) and a single pathogen detection in the other two cases (Case 2 and 5).

In Case 7 and 14, the culture results suggested a single infection, and mNGS complemented the diagnosis of polymicrobial PJI. In Case 7, a sinus tract infection was present at the incision and the culture results showed the pathogenic microorganism was *Enterococcus faecalis*, while mNGS analysis indicated *Enterobacter cloacae* and *Acinetobacter pittii* infection. Case 14 had a sinus tract infection, and the culture result indicated *Streptococcus oralis* infection, while mNGS detected *Streptococcus oralis* and supplemented the diagnosis of *Klebsiella oxytoca* infection. 

In Case 2 and 5, both culture and mNGS suggested monomicrobial detection, but the combination of results confirmed the diagnosis of polymicrobial PJI. Case 2 was associated with a *Mycoplasma* urinary tract infection; *Candida tropicalis* was identified by culture, while mNGS complemented the detection of *Mycoplasma hominis*. Case 5 was also found to have a *Mycoplasma* urinary tract infection; culture identified a *Staphylococcus aureus* infection, and mNGS reported a *Mycoplasma hominis* infection. 

In summary, for cases with a single infection identified by conventional microbial culture, mNGS complemented the diagnosis of polymicrobial PJI.

### 3.8. Cases with Multiple Pathogens Detected by Both Culture and mNGS 

Among eight cases with a positive polymicrobial culture (Case 1, 4, 6, 8, 9, 10, 11 and 13), the mNGS results of Case 8 were completely consistent with culture; the results of Case 11 were completely inconsistent; the remaining six cases (Case 1, 4, 6, 9, 10 and 13) were partially consistent.

In Case 8, both culture and mNGS results identified *Enterococcus faecalis* and *Staphylococcus epidermidis* infections. In Case 11, the culture results were positive for *Klebsiella pneumoniae*, *Escherichia coli* and *Clostridium ramosum*, while the mNGS results were *Prevotella bivia*, *Streptococcus constellatus* and *Dialister invisus*. In Case 1, both culture and mNGS results indicated *Candida parapsilosis* infection; in addition, culture results suggested *Staphylococcus haemolyticus* and *Staphylococcus epidermidis* infections, but mNGS did not detect the relevant pathogens. Instead, the use of this method resulted in a diagnosis of *Cutibacterium acnes* infection. In Case 4, culture results showed *Staphylococcus epidermidis*, *Enterococcus faecalis*, *Acinetobacter nosocomialis* and *Acinetobacter baumannii* infection; mNGS detected all of the above pathogens and added the diagnosis of *Mycobacterium tuberculosis*. In Case 6, the culture results suggested a polymicrobial infection of *Finegoldia magna* and *Staphylococcus epidermidis*; mNGS confirmed the diagnosis of *Finegoldia magna* but failed to detect *Staphylococcus epidermidis*. Instead, it resulted in a diagnosis of *Anaerococcus tetradius* and *Peptoniphilus_lacrimalis* infection. In Case 9, culture results indicated a polymicrobial infection of *Klebsiella pneumoniae* and *Bacteroides fragilis*; mNGS confirmed the culture results and added the diagnosis of *Clostridium clostridioforme* infection. In Case 10, both mNGS and culture results indicated the presence of *Staphylococcus epidermidis* and *Escherichia coli*, in addition, mNGS diagnosed *Shigella boydii* infection. In Case 13, mNGS and culture methods both detected *Staphylococcus epidermidis* infection; in addition, *Ralstonia pickettii* infection was identified by culture, while mNGS supplemented the diagnosis of *Cutibacterium acnes* infection.

In conclusion, mNGS played an important role, even in cases where conventional culture results suggested polymicrobial infection. In most cases, mNGS can unbiasedly detect common and rare pathogens without any prior hypothesis.

## 4. Discussion

Our previous studies have shown that mNGS has a high diagnostic efficacy for PJI diagnosis [27,30,32,37,38]. Several studies have reported consistent results [26,39,40]. In this study, we further explored the effectiveness of mNGS for the pathogenic diagnosis of polymicrobial PJI based on previous studies. Our results were similar to those of Wang et al., who studied the application of mNGS for mixed pulmonary infections diagnosis [28]. The sensitivity of mNGS in diagnosing polymicrobial PJI was higher than that of conventional microbial culture (85.7% vs. 57.1%), but the specificity was lower compared with culture (60% vs. 100%).

According to the results of several multicenter studies, the incidence of polymicrobial PJI is 19% to 36% [3,5,6,7,41]. Traditional molecular diagnostic techniques such as PCR require pre-defined targets to design primers for detection, and the diagnostic performance for PJI is not high [38,42]. mPCR allows for multiple targets to be set, on the basis of traditional PCR. However, studies have shown that mPCR was superior to culture in detecting low-virulent pathogens such as coagulase-negative staphylococci, but for the detection of high-virulent pathogens such as *Staphylococcus aureus* and *Streptococcus*, was less efficient than culture; the overall performance of mPCR was comparable to culture [43]. In addition, the time needed for the detection of microorganisms varies between testing techniques, which ultimately affects treatment decisions. For acute PJI (4 weeks from symptoms), debridement, antibiotics, and implant retention can be used [44], while, after the infection becomes chronic, after more than 4 weeks of symptoms, most options are stage I or stage II exchange [45]. The average turnaround times (TATs) for conventional bacterial cultures take 3 days, compared to 7 days for fungi and 45 days for mycobacteria [23]. In contrast, multiplex PCR is able to detect pathogenic bacteria within 2 h [21], and the typical mNGS time from sample receipt to the final result is 48 h, while it can be reduced to 6 h with nanopore-sequencing technology [23].

Our study showed that mNGS was more efficient and it can unbiasedly detect high- and low-virulence pathogens, which was an advantage that neither mPCR nor culture had. In this study, polymicrobial PJIs were diagnosed in 14 (20.3%) of 69 PJI patients, and it is noteworthy that if conventional microbial cultures were applied alone, six patients would have been missed, including two culture-negative patients and four mono-culture-positive patients. The inadequate diagnosis of polymicrobial infections and inappropriate postoperative antibiotic therapy ultimately lead to the failure of prosthetic revision surgery. The empirical use of broad-spectrum antibiotics in culture-negative patients not only wastes medical resources and increases patient’s financial burdens, but also increases the incidence of antibiotic-related complications [46]. In clinical practice, the diagnosis of polymicrobial PJIs by conventional culture methods may result in an inadequate diagnosis of polymicrobial infections, due to factors such as preoperative antibiotic use [47], and the competitive inhibition effects of polymicrobial bacteria during culture [12]. This can be addressed by using mNGS to improve the detection rate of pathogens and to complement the diagnosis of polymicrobial infections.

Although mNGS can improve detection efficiency, the application of mNGS alone can also lead to false positives. In this study, of 69 patients diagnosed with PJI by 2018 ICM diagnostic criteria, multiple infections were detected by mNGS in 34 cases, and 22 of them were identified as false positives. As some of the pathogens detected in these cases, such as *Citrobacter freundii* and *Ureaplasma urealyticum*, were not the reported causative agents of osteoarticular infections, the cultures results remained negative after special cultures and extended cultures for 14 days, no antibiotic treatment was selected for this pathogen, and the patient prognosis was good.

For most cases, mNGS reported the pathogens detected by conventional microbial culture, but there were also a few cases where mNGS failed to detect the cultured pathogens, resulting in false-negative results. In Cases 2 and 5, mNGS only detected *Mycoplasma hominis*, while *Candida tropicalis* (Case 2) and *Staphylococcus aureus* (Case 5) were not identified, which may be because the patient received preoperative antibiotic therapy. However, it has been reported that the detection sensitivity of mNGS is significantly higher than that of conventional culture in patients with previous antibiotic exposure [25]. However, it should be noted that with the prolongation of antibiotic treatment time, the detection rate of pathogenic microorganisms by mNGS showed a decreasing trend [48].

In summary, mNGS has obvious advantages in the diagnosis of periprosthetic polymicrobial infection, due to its excellent pathogen detection efficiency. However, there are still many false positives when using mNGS alone. Therefore, the combined results of mNGS and culture should be considered when diagnosing polymicrobial infection. When mNGS and culture detect multiple pathogens, clinicians and microbiologists should be consulted. When diagnosing, we believe that when the detection results of the two pathogens are consistent, the pathogenic microorganism can be basically determined; if they are inconsistent, the source of the pathogen should be judged according to the medical history, especially regarding some characteristics such as oral infections, urinary tract infections, beriberi and other infections of the body. For example, in Cases 2 and 5, the mNGS and culture results were inconsistent. According to a patient’s history of urinary tract infection, the *Mycoplasma hominis* detected by mNGS was confirmed as the infectious agent, so the diagnosis of polymicrobial PJI was made. In addition, the pathogenicity of the pathogen itself must also be considered. For example, pathogens such as *Citrobacter freundii*, *Pseudomonas monteilii*, and *Malassezia globosa* have not been reported to cause bone and joint infections; if they appear in the test results, and the number of reads is not high, a pathogenic diagnosis should be carefully considered.

Due to the high price of mNGS, and based on the experience of our clinic, it is recommended to combine different testing methods for patients with the following conditions. (1) Patients with a long history of infection and combined sinus tract infection, mPCR, and culture can be performed at the same time, then medication can be administered according to the results of mPCR and then adjusted according to the culture, and if the treatment is still not effective, further sexual mNGS testing can be performed. (2) For cases with positive culture results, with species such as *Enterococci* and Gram-negative bacilli, which are often reported as polymicrobial infections, there is a greater chance of mixed infections, and mNGS testing can be performed directly. (3) In cases with negative culture, mPCR testing can be performed first, and if still negative, further mNGS testing can be performed.

This study also has several limitations. Firstly, due to the low incidence of PJI, this study only involved a single center, the sample size was far from adequate, and further expansion of the sample size in combination with multiple centers is needed to verify the true clinical value of the technique. Secondly, there is no uniform standard for the diagnosis of polymicrobial PJIs, currently. Although orthopedic experts and microbiologists can make a diagnosis of polymicrobial PJI based on the results of culture, next-generation sequencing, and their corresponding standards, the deviation caused by the misclassification of some cases cannot be avoided. Finally, we did not compare the sensitivity and specificity of mNGS for different sample types.

Overall, orthopedic surgeons will benefit more and more from this technique in the future, but culture should still be considered the gold standard for PJI diagnosis, as indicated in the International Consensus Meeting (ICM) [49] and European Bone and Joint Infection Society (EBJIS) [50] criteria. Once the issue of the high numbers of false-positive results produced by the mNGS test is resolved, mNGS may help us in the future to refine guides for PJI and polymicrobial PJI diagnosis.

## 5. Conclusions

This study shows that mNGS can improve the diagnosis of polymicrobial PJI. Culture combined with mNGS is a promising method in detection. It is worth noting that when the mNGS results are inconsistent with the culture results, the pathogen should be judged according to the clinical history, and then the appropriate antibiotic treatment plan should be selected.

## Figures and Tables

**Table 1:** Demographic characteristics of included patients.

Characteristics	All Patients (n = 91)	PJI (n = 69)	AF (n =22)	*p*-Value
Age, years, median (range)	64.6 ± 13.5	65.3 ± 13.5	62.7 ± 13.6	0.448
Gender, female, n (%)	53 (58.2%)	42 (60.9%)	11 (50.0%)	0.368
Location, n (%)				0.099
Hip	46 (50.5%)	30 (43.5%)	14 (63.6%)	
Knee	45 (49.5%)	39 (56.5%)	8 (36.4%)	
Sinus tract, n (%)	15 (16.5%)	15 (21.7%)	0 (0.0%)	0.017
Antibiotics prior to surgery, n	23	23	0	0.002
ESR, mm/h	53.3 ± 37.4	65.6 ± 33.8	14.5 ± 14.6	<0.001
CRP, mg/L	44.2 ± 43.6	51.8 ± 46.4	20.2 ± 19.4	0.002
SF-WBC × 106/L	21,976.9 ± 44,518.9	28,471.2 ± 49,456.3	1608.4 ± 797.9	<0.001
SF-PMN, %	73.8 ± 47.4	75.4 ± 16.0	68.9 ± 93.7	<0.001

CRP: C-reactive protein, ESR: erythrocyte sedimentation rate, SF-WBC: synovial fluid white blood cell count, SF-PMN: synovial fluid polymorphonuclear cell.

**Table 2:** Comparison of the diagnostic efficiency of PJI between culture and mNGS.

Methods	No. of Patients (n =)	PJI Group	AF Group	Sensitivity % (95% CI)	Specifificity % (95% CI)	PPV % (95% CI)	NPV % (95% CI)	Accuracy (95% CI)
Intraoperative sample culture	91	49/20	1/21	71.0 (58.8–81.3)	95.4 (77.1–99.8)	98.0 (89.3–99.9)	51.2 (35.1–67.1)	76.9 (66.9–85.1)
Intraoperative sample mNGS	91	63/6	3/19	91.3 (82.0–96.7)	86.3 (65.0–97.0)	95.4 (87.2–99.0)	76.0 (54.8–90.6)	90.1 (82.0–95.3)

PPV: positive predictive value, NPV: negative predictive value, CI: confidence interval.

**Table 3:** Comparison of the diagnostic efficiency of polymicrobial PJI between culture and mNGS.

Methods	No. of Patients (n =)	Polymicrobial PJI Group	Mono-Microorganism PJI Group	Sensitivity % (95% CI)	Specifificity % (95% CI)	PPV % (95% CI)	NPV % (95% CI)	Accuracy (95% CI)
Intraoperative sample culture	69	8/6	0/55	57.1 (28.9–82.3)	100 (93.5–100)	100 (63.0–100)	90.2 (79.8–96.3)	91.3 (82.0–96.7)
Intraoperative sample mNGS	69	12/2	22/33	85.7 (57.2–98.2)	60.0 (45.9–73.0)	35.3 (19.8–53.5)	94.3 (80.8–99.3)	65.2 (52.8–76.3)

Abbreviations: PPV: positive predictive value, NPV: negative predictive value, CI: confidence interval.

**Table 4:** Cases clinically diagnosed as polymicrobial PJI.

Patient No.	Administration of Antibiotic Pre-Operatively (Yes, Y/No, N)	Underlying Joint Disorder	Sinus Tract OR Incision Reputure	Co-Morbidity	Infection Type	mNGS Results	Culture Results	Antibiotic Regimen
No. 1	Y	Osteoarthritis	none	Hypertension; Diabetes mellitus	chronic	*Cutibacterium acnes*; *Candida parapsilosis*	*Staphylococcus haemolyticus*; *Staphylococcus epidermidis*; *Candida parapsilosis*	*Vancomycin*; *Moxifloxacin*; *Fluconazole*
No. 2	Y	femoral-head necrosis	none	Hypertension; urinary tract infection	acute	*Mycoplasma hominis*	*Candida tropicalis*	*Fluconazole*; *Erythromycin*
No. 3	Y	Osteoarthritis	none	None	chronic	*Mycoplasma hominis*; *Acinetobacter_baumannii*	*Negative*	*Vancomycin*; *Ceftazidime*; *Levofloxacin*; *Doxycycline*
No. 4	Y	Septic Arthritis	Sinus tract	None	chronic	*Enterococcus faecalis*; *Staphylococcus epidermidis*; *Acinetobacter nosocomialis*; *Acinetobacter baumannii*; *Mycobacterium tuberculosis*	*Enterococcus faecalis*; *Staphylococcus epidermidis*; *Acinetobacter nosocomialis*; *Acinetobacter baumannii*	*Vancomycin*; *Imipenem*; *Levofloxacin*; *Rifampicin*; *isoniazid*
No. 5	Y	Osteoarthritis	none	Diabetes mellitus; urinary tract infection	acute	*Mycoplasma hominis*	*Staphylococcus aureus*	*Vancomycin*; *Cefuroxime*; *Erythromycin*
No. 6	Y	Osteoarthritis	incision reputure	None	chronic	*Finegoldia magna*; *Anaerococcus tetradius*; *Peptoniphilus_lacrimalis*	*Finegoldia magna*; *Staphylococcus epidermidis*	*Vancomycin*; *Meropenem*; *Piperacillin*; *Linezolid*; *Amoxicillin*; *Metronidazole*
No. 7	N	Osteoarthritis	Sinus tract	Hypertension; gout	acute	*Enterobacter cloacae*; *Acinetobacter pittii*	*Enterococcus faecalis*	*Meropenem*; *Vancomycin*; *Piperacillin*; *Penicillin*; *Teicoplanin*
No. 8	N	Osteoarthritis	none	none	chronic	*Enterococcus faecalis*; *Staphylococcus epidermidis*	*Enterococcus faecalis*; *Staphylococcus epidermidis*	*Vancomycin*; *levofloxacin*;
No. 9	Y	femoral-head necrosis	none	Hypertension; Diabetes mellitus; Hypoproteinemia	chronic	*Klebsiella pneumoniae*; *Bacteroides fragilis*; *Clostridium clostridioforme*	*Klebsiella pneumoniae*; *Bacteroides fragilis*	*Vancomycin*; *Meropenem*; *Colapitol*
No. 10	Y	Fracture	none	Diabetes mellitus	chronic	*Staphylococcus epidermidis*; *Escherichia coli*; *Shigella boydii*	*Staphylococcus epidermidis*; *Escherichia coli*	*Vancomycin*; *Meropenem*
No. 11	Y	Fracture	none	Diabetes mellitus	chronic	*Prevotella bivia*; *Streptococcus constellatus*; *Dialister invisus*	*Klebsiella pneumoniae*; *Escherichia coli*; *Clostridium ramosum*	*Vancomycin*; *Meropenem*; *Ceftazidime*; *Metronidazole*
No. 12	N	Osteoarthritis	Sinus tract	Hyperthyroidism; Diabetes mellitus	chronic	*Pseudomonas aeruginosa*; *Klebsiella oxytoca*; *Mycoplasma hominis*	*Negative*	*Vancomycin*; *Meropenem*; *Ceftazidime*; *Doxycycline*
No. 13	N	Osteoarthritis	none	None	chronic	*Staphylococcus epidermidis*; *Cutibacterium acnes*	*Ralstonia pickettii*; *Staphylococcus epidermidis*	*Vancomycin*; *Linezolid*
No. 14	Y	Osteoarthritis	Sinus tract	Diabetes mellitus; Rheumatoid arthritis	chronic	*Streptococcus oralis*; *Klebsiella oxytoca*	*Streptococcus oralis*	*Vancomycin*; *Meropenem*; *Ceftriaxone*

## Data Availability

The original contributions presented in the study are included in the article, further inquiries can be directed to the corresponding authors.
